# Human Organoids for Predictive Toxicology Research and Drug Development

**DOI:** 10.3389/fgene.2021.767621

**Published:** 2021-11-01

**Authors:** Toshikatsu Matsui, Tadahiro Shinozawa

**Affiliations:** Drug Safety Research and Evaluation, Research, Takeda Pharmaceutical Company Limited, Fujisawa, Japan

**Keywords:** organoid research, liver organoid, cardiac organoid, kidney organoid, intestinal organoid, brain organoid, predictive toxicology, drug development

## Abstract

Organoids are three-dimensional structures fabricated in vitro from pluripotent stem cells or adult tissue stem cells *via* a process of self-organization that results in the formation of organ-specific cell types. Human organoids are expected to mimic complex microenvironments and many of the *in vivo* physiological functions of relevant tissues, thus filling the translational gap between animals and humans and increasing our understanding of the mechanisms underlying disease and developmental processes. In the last decade, organoid research has attracted increasing attention in areas such as disease modeling, drug development, regenerative medicine, toxicology research, and personalized medicine. In particular, in the field of toxicology, where there are various traditional models, human organoids are expected to blaze a new path in future research by overcoming the current limitations, such as those related to differences in drug responses among species. Here, we discuss the potential usefulness, limitations, and future prospects of human liver, heart, kidney, gut, and brain organoids from the viewpoints of predictive toxicology research and drug development, providing cutting edge information on their fabrication methods and functional characteristics.

## Introduction

Drug-induced adverse drug reactions are one of the leading causes for the discontinuation of drug development projects and the withdrawal of drugs from the market (Cook et al., 2014). It is, thus, essential to minimize adverse reactions to improve patient safety. For pharmaceutical companies, the decision to discontinue drug development due to adverse reactions at an early stage leads to a reduction in development costs and human resources. Therefore, studies focused on accurately predicting potential drug-induced adverse effects in humans in the early stages of drug development are important (Fitzpatrick, 2020). Toxicity studies have previously relied on animals, but species differences in anatomy, function, and morphology in various tissues have hampered the accurate understanding of the mechanisms of toxicity and the acquisition of accurate predictions regarding toxicity in humans (Van Norman, 2019; 2020). Additionally, animal studies are expensive and time-consuming, which can limit the number of substances tested. Therefore, *in vitro* assays using human cells are required to overcome these problems and to align with the concept of the 3Rs (replacement, reduction, and refinement).

Organoids are miniature organs prepared from adult tissue-resident stem cells (ASCs) or pluripotent stem cells (PSCs) *in vitro* (Bartfeld and Clevers, 2017). They are formed as three-dimensional (3D) tissue-like structures through the self-organization, self-renewal, and differentiation capacity of the stem cells (Fatehullah et al., 2016). Compared to the conventional two-dimensional (2D) cultured cells or spheroids, organoids are anatomically and functionally close to the organs in the living body (Kim et al., 2020), and they can be constructed from both normal and diseased tissues. Thus, various research applications using human organoids are expected, not only for basic research such as embryology, physiology, and evolution but also to help understand disease conditions and for drug discovery research, such as drug efficacy and toxicity (Kim et al., 2020). Additionally, organoids hold great promise for regenerative medicine (Rodrigues and Banales, 2021).

In this review, we described drug-induced organ toxicities in five areas or organs (liver, heart, kidneys, gastrointestinal tract, and brain); additionally, the *in vitro* predictive assays using human corresponding cells that have been developed so far are also mentioned. Furthermore, we present state-of-the-art examples of toxicity studies using 3D human organoids produced from human stem cells, which have received growing attention in recent years, and expand on their limitations and prospects. The cell sources, fabrication methods, and characteristics of these 3D organoids are described in detail in each subsequent section and summarized in Table 1 at the end of this article. The test drugs used and the toxic endpoints measured in these organoid studies are also included in the Table 1.

**TABLE 1:** Organoid-based toxicity assessment.

Organoid type	Organoid sources	Fabrication methods	Characteristics	Test substances	Toxic endpoints	References
Liver organoid	ESCs	Differentiation of ESCs into foregut endoderm progenitor populations that are further maturated into hepatic progenitor cells by the WNT signaling pathway, then cultured under 3D suspension culture conditions	By selecting the size of the organoids, it is possible to produce a more complex structure, including cholangiocytes and stromal cells, in case of large organoids	Acetaminophen	Viability (WST-1)	Sgodda et al. (2017)
	Primary human hepatocytes, hepatic stellate cells, and Kupffer cells	Produced with a mixture of 80% hepatocytes, 10% hepatic stellate cells, and 10% Kupffer cells and seeded onto non-adherent, round-bottomed plates	Composed of a representative population of cells *in vivo*, capable of maintaining high viability over a long period of time, and is metabolically active, able to respond to and metabolize drugs accurately	Environmental toxins (glyphosate, lead, mercury, thallium)	Viability (ATP and live cell staining)	Forsythe et al. (2018)
	Human liver tissue	Isolation of duct cells from human liver tissue by chopping and enzyme digestion, and embedding of the isolated duct cells in Matrigel followed by the addition of differentiation factors	Shows the characteristics of hepatocytes rather than HepG2 cells such as positive nuclear staining of HNF4A, clear positive glycogen accumulation, and a more stable albumin secretion	PL-inducing drugs (amikacin, amiodarone, sertraline, acetaminophen)	Viability (ATP), morphological changes, albumin secretion, expression of genes related to phospholipidosis	Lee et al. (2020)
	iPSCs	Differentiation of iPSCs into foregut, dissociation into single cells, and culture in a specialized medium to enhance organoid formation, followed by harvesting and resuspension in Matrigel on the Ultra-Low Attachment Multiwell Plate	Exhibits properties that include bile transport functions, allows assessment of susceptibility based on polymorphism at organoid resolution, and is amenable to high-throughput toxicity screening	238 marketed drugs including 206 DILI compounds and 32 non-DILI compounds	Biliary excretion capacity, cell viability (ATP), and mitochondrial membrane potential	Shinozawa et al. (2021)
Cardiac organoid	iPSCs and fibroblasts	Cell suspension consisted of 90% iPSC-derived CMs; 10% cardiac fibroblasts were suspended in a hydrogel, seeded in a specialized culture platform microwell (Biowire™ II), and subjected to a 10-weeks electrical field stimulation to promote maturation	Shows positive force frequency and post-rest potentiation and improved sarcomeric organization and is enriched for gene expression patterns of the corresponding adult human heart cardiac regions, enables polygenic cardiac disease modeling	6 cardioactive drugs (Zhao et al.), 12 cardioactive drugs (Feric et al.) and 8 reported positive inotropes (Qu et al.)	Contractility, Calcium transients, and gene expression related to cardiotoxicity pathway	Zhao et al. (2019), Feric et al. (2019),Qu et al. (2020)
	ESCs and fibroblasts	Cell suspension consisted of 90% ESC-derived CMs, and 10% dermal fibroblasts were re-suspended in aqueous scaffold materials (Matrigel and collagen I solution) in a dedicated bioreactor, followed by gelation and self-assembly during incubation to fabricate a miniature cardiac organoid that mimics a human ventricle	Shows organized sarcomeres with myofibrillar microstructures, upregulation of key genes related to calcium-handling, ion channel, and cardiac-specific proteins and allows direct measurement of stroke volume, EF, cardiac output, pressure volume loop, and so on	6 cardioactive drugs (Li et al.) and 25 cardioactive drugs (Keung et al.)	Contractility	Li et al., 2018, Keung et al., 2019
Cardiac organoid	iPSCs, hCMECs, and hCFs	Cell suspensions of iPSC-derived CMs, hCMECs, and hCFs in a ratio of 4:2:1 were seeded in ultra-low attachment spheroid microplates	Exhibits typical morphological and cellular compositional characteristics similar to those present in myocardium and are amenable to high-throughput toxicity screening	15 FDA approved structural cardiotoxins and 14 FDA approved nonstructural cardiotoxins	Viability (ATP), endoplasmic reticulum integrity, and mitochondrial membrane potential	Archer et al. (2018)
	iPSCs-CMs, hCFs, HUVECs, and hADSCs	A cell suspension composed of 50% iPSC-derived CMs and 50% non-myocytes (at a 4:2:1 ratio of FBs: HUVECs: hADSCs) was seeded into agarose hydrogel molds and submerged with culture medium, followed by incubation under hypoxic conditions and treatment with noradrenaline	Mimics the characteristics of myocardial infarction, including pathological metabolic changes, fibrosis and altered calcium handling, at transcriptomic, structural, and functional levels	doxorubicin	Contractility and apoptosis (TUNEL staining)	Richards et al. (2020)
Kidney organoid	ESCs and iPSCs	Differentiation of 3D cultured PSC-derived cavitated spheroids into segmented, nephron-like kidney organoids by GSK3b inhibition to result in cell population with proximal tubular, podocyte, and endothelial characteristics	3D culture system that reconstitutes functional, structured epithelia modelling the epiblast, kidney tubular cells, and podocyte-like cells	cisplatin and gentamicin	Expression of kidney injury biomarker (Kim-1)	Freedman et al. (2015)
	ESCs and iPSCs	PSC-derived intermediate mesoderm was treated with GSK-3b inhibitor and FGF9 to differentiate into nephron progenitor cells of metanephric mesenchyme and to further induce pretubular aggregates and renal vesicles	Contains epithelial nephron-like structures expressing markers of podocytes, proximal tubules, loops of Henle, and distal tubules in an organized, continuous arrangement	gentamicin and cisplatin	Expression of kidney injury biomarker (Kim-1)	Morizane et al. (2015)
	iPSCs	Monolayer culture of PSC-derived posterior primitive streak to induce intermediate mesoderm, followed by a 3D culture to promote self-organizing nephrogenic events leading to organoid formation	Contains nephrons associated with a collecting duct network surrounded by renal interstitium and endothelial cells	cisplatin	Apoptosis (caspase-3)	Takasato et al. (2015)
	Renal fibroblasts, huvec, and RPTECs	Renal fibroblasts and huvec were combined in a 50:50 ratio and seeded into a microwell plate using a 3D bioprinter, followed by the addition of RPTECs	Shows formation of extensive microvascular networks and tight junctions and expression of renal uptake and efflux transporters	cisplatin	Viability (LDH release) and expression of genes related to fibrosis (TGF beta)	King et al., 2017
Intestinal organoid	Ileal small intestinal tissue	Digested human primary ileal tissue, isolated intestinal crypts by passing the minced tissue sections through filter mesh, and suspended in Matrigel and cultured in a specialized medium	Enteroids collected with a mean diameter of 600 μm indicate the presence of Paneth cells, enteroendocrine cells, goblet cells, and enterocytes	31 marketed diarrheagenic and non-diarrheagenic drugs	Viability (ATP)	Belair et al. (2020)
	fibroblasts and primary small intestinal epithelial cells	Generated by seeding small intestinal epithelial cells on a supportive layer of fibroblasts under air-liquid interface conditions in a transwell format	Composed of a monolayer of simple columnar epithelial cells with basally positioned nuclei, consistent with enterocytes	39 marketed drugs with different diarrhea-genic risk	Barrier function and Cell proliferation (MTT assay)	Peters et al. (2019)
Brain organoid	iPSCs	Cultured embryoid bodies generated from iPSC were embedded in Matrigel and seeded onto plates in organoid differentiation media to form cerebral organoids and then transferred to a spinner platform for long-term culture	Exhibits similar developmental. Patterns and contains multiple types of brain cells (NSCs, neurons, and astrocytes), and distinct multi-layered, cortical-like neuronal zone and choroid plexus	Ethanol	Caspase 3 activity and functional and morphological alterations of mitochondria	Arzua et al. (2020)
	ESCs	ESC-derived cerebral organoids generated by adding dual SMAD-signaling inhibitors to neural induction medium to promote neuroepithelial expansion and recapitulate fetal cerebral development *in vitro*	Displays the features of brain development, including the expression of neural stem cells, neurons, and astrocyte markers, indicating cerebral organoid stages up to early fetal stage	acrylamide	Apoptosis, expression of genes related to the NRF2 pathway, and tau phosphorylation	Bu et al. (2020)
	iPSCs	Embryoid bodies differentiated from iPSC were cultured in a selected growth factors and ECM culture conditions to generate neuroectoderm, followed by development into neural epithelial tissue, then plated and incubated on an orbital shaker to form cerebral organoids	Exhibits complex morphology and neuron-specific cell differentiation and expresses marker genes related to forebrain, hippocampus, hindbrain, and prefrontal cortex	vincristine	Caspase 3 activity	Liu et al. (2019)
	iPSCs	Neural progenitor cells differentiated from iPSC were differentiated into brainspheres by incubating in a dedicated differentiation medium under constant gyratory shaking for up to 8 weeks	Expresses mature neuronal markers (NF200, βIII-tubulin and MAP2), astrocytic marker GFAP, and oligodendrocyte marker O1, indicating that diverse neuronal and glial cell populations are present	rotenone	Reactive oxygen species (ROS) and mitochondrial function	Pamies et al. (2018)

## Liver Organoids and the Prediction of Hepatotoxicity

### Overview

Drug-induced hepatotoxicity is a serious problem (Hoofnagle and Bjornsson, 2019). It reduces the probability of clinical success and is a major reason for drug withdrawal from the market (Cook et al., 2014). Additionally, it causes acute liver injury (Reuben et al., 2010) and ranks high in the list of drug-induced adverse reactions that occur in the clinical setting. Although animal studies have been conducted to predict the risk of drug-induced hepatotoxicity, it has been reported that, owing to species differences, only 40–50% of clinical hepatotoxicity can be predicted (Olson et al., 2000). There has, consequently, been a focus on the development of highly predictive assays for drug-induced hepatotoxicity and various *in vitro* assays using human hepatic cells. To date, 2D cultured HepG2 (Tolosa et al., 2012), HepaRG, primary human hepatocytes (PHH), and PSC-derived hepatocyte-like cells (Lu et al., 2015b) have been utilized to evaluate toxicity. However, HepG2 is a human hepatoma cell line that does not express cytochrome P450 enzymes (Guo et al., 2011). HepaRG is another differentiated hepatic cell line, but it expresses various CYPs, as opposed to HepG2, and has thus been used for high-throughput toxicity screening (Saito et al., 2016). However, HepaRG do not produce detectable levels of urea (Lubberstedt et al., 2011). PHH is considered the gold standard hepatic cell model (Gomez-Lechon et al., 2014); however, it rapidly undergoes dedifferentiation upon 2D culture (Elaut et al., 2006), loses metabolic activity, and does not proliferate *in vitro*, making it difficult to obtain a large amount of PHH for large-scale screening. Inter-donor functional variability also exists in PHH (den Braver-Sewradj et al., 2016). Human PSCs have attracted increasing attention for their ability to proliferate indefinitely and differentiate into hepatocyte-like cells *in vitro*. However, hepatocyte-like cells derived from human PSCs have an immature phenotype (Schwartz et al., 2014; Baxter et al., 2015). Furthermore, the liver is composed of several cell types, including cholangiocytes, stellate cells, Kupffer cells, and liver sinusoidal endothelial cells, in addition to hepatocytes. Each of these cell types possesses unique functions that cooperatively regulate the physiological hepatic functions (Trefts et al., 2017). Based on the facts mentioned above, it is possible that the current 2D cultured hepatocytes including the gold standard hepatic model PHH do not fully recapitulate physiological liver function, and the role of non-hepatocytes should be considered in future disease modeling, drug screening, and toxicity assessments.

To overcome these limitations, scientists have struggled to create a more physiologically relevant *in vitro* hepatic model. In 2015, Huch et al. succeeded in producing functional 3D hepatic organoids that were differentiated from ductal cells that had been extracted from cell suspensions obtained through the collagenase-mediated digestion of human liver biopsy tissues, and showed that the expanded cells that had differentiated from the adult bile duct-derived cells had preserved their genetic integrity after being cultured for a period of months (Huch et al., 2015). Takebe et al. first reported the creation of hepatic organoids by co-culturing human iPSCs-derived hepatic endoderm cells, human umbilical vein endothelial cells (HUVECs), and mesenchymal cells (Takebe et al., 2013). The resulting liver bud organoids developed into hepatic tissues, showing mature hepatic features upon transplantation into mice. They also established a method to produce liver organoids from endodermal, endothelial, and mesenchymal progenitor populations specified entirely from human iPSCs (Takebe et al., 2017). These liver organoids that were differentiated from either human ASCs or PSCs are expected to fill the gap between 2D human cell cultures and animal models because they are more physiologically relevant to adult liver tissue and can thus be utilized for liver disease modeling, regenerative medicine, and drug screening. Indeed, there have been several studies using human liver organoids for toxicological evaluations. Sgodda et al. developed a scalable 3D suspension culture system in which human ESCs-derived hepatic cells could be maintained for up to 3 weeks (Sgodda et al., 2017). They differentiated human ESCs into hepatic progenitors by modulating the WNT signaling pathway and transferred them to a 3D culture system to enhance organoid formation. The fabricated ESC-derived 3D hepatic organoids were more sensitive to acetaminophen-induced toxicity than 2D cultured ESC-derived hepatic cells. Forsythe et al. produced human liver organoids by embedding a cell suspension composed of 80% primary human hepatocytes, 10% hepatic stellate cells, and 10% Kupffer cells into Matrigel, and seeded them into non-adherent, round-bottom, 96-well plates to form aggregates and produce spherical organoids (Forsythe et al., 2018). They screened four environmental heavy metals using the fabricated 3D hepatic organoids and examined changes in the ATP content as a toxicity readout. All the tested substances showed dose-dependent toxicity and proposed the utility of 3D organoids for toxicity screening applications. Lee et al. created 3D hepatic organoids to predict drug-induced phospholipidosis (PL). They isolated duct cells from human liver tissue and embedded them in Matrigel, followed by the addition of differentiation factors to create the organoids (Lee et al., 2020). They, then, compared the effects of three marketed drugs known to cause PL in both 3D hepatic organoids and conventional 2D cultured HepG2 cells, by analyzing the differences in function, morphology, and gene expression between the two assay systems. They found that the 3D cultured hepatic organoids were more sensitive to drug-induced PL. The above-mentioned reports examined only a few test substances for toxicity evaluation using 3D organoids. However, Shinozawa et al. reported the development and validation of high-throughput toxicity screening using hepatic 3D organoids. They fabricated self-organized human liver organoids that had human hepatocyte-like properties, including bile transport function, by embedding foregut cells differentiated from human iPSCs into Matrigel (Shinozawa et al., 2021). After confirming organoid formation, they added maturation factors and replated the embedded organoids in floating cultures and seeded them onto 384-well plates for high-throughput live imaging. Using this methodology, they evaluated 238 pharmaceuticals, including 32 negative controls and 206 reported DILI compounds, using bile acid transport activity and cell viability as readouts. The results revealed high predictivity, with 88.7% sensitivity and 88.9% specificity. They also demonstrated that bosentan-induced cholestasis is specific to CYP2C9*2 HLO, suggesting that different susceptibilities based on the polymorphism can be observed using human liver organoids.

### Limitations and Future Perspectives

Studies on the prediction of drug-induced hepatotoxicity using human 3D liver organoids are still limited, but they are expected to increase in the future, as they are attracting increasing attention. From a practical point of view, it is important to compare the predictability of drug-induced hepatotoxicity in conventional 2D cultures and complex 3D organoid culture systems using a wide array of drugs. Such large-scale validation studies will help us gain a better understanding of drugs/MOA that are detectable or not detectable, depending on the different hepatic models. Another point to consider is the immaturity of 3D liver organoids, as it has been reported that PSC-derived hepatocytes express immature or fetal markers (Harrison et al., 2021) and do not have all the functions of mature hepatocytes. Various approaches have been proposed to address this issue, including the overexpression of transcription factors or miRNAs and the addition of growth factors or small molecules to promote the differentiation and maturation of hepatocytes (Chen et al., 2018). Assay systems using human hepatocytes, which more closely resemble the functions of adult liver tissue, could help predict potential drug-induced hepatotoxicity more accurately in humans. Robust assay reproducibility among laboratories is also necessary for the adoption of organoid-based toxicity screening during early drug development.

## Cardiac Organoids and the Prediction of Cardiotoxicity

### Overview

Drug-induced cardiotoxicity is the leading cause of drug attrition during the development process (Cook et al., 2014). Various categories of drugs are known to cause prolonged QT and torsades de pointes (TdP), a life-threatening ventricular arrhythmia (Roden, 2016). In addition, many cancer drugs have been reported to cause reduction in the left ventricular ejection fraction (LVEF), and their long-term use is associated with an increased risk of developing chronic heart failure (Kerkela et al., 2006; Chu et al., 2007; McGowan et al., 2017). These factors contribute to their discontinuation during drug development (Ferri et al., 2013) and are clinically problematic (Hall et al., 2013; Roden, 2016). Both rodents and larger animals are used to monitor cardiac function *via* ECG or echocardiography to determine whether a candidate drug causes alterations in cardiac function during the later phases of development. However, because of the differences in cardiac function among species, the labor-intensive and time-consuming nature of animal experiments, and due to considerations for the 3Rs, there is a growing demand to develop an assay system that can predict drug-induced cardiotoxicity in humans with a high accuracy during the early stages of drug development. Cell-based functional and structural assays have been used as *in vitro* prediction tools for drug-induced cardiotoxicity. In particular, the binding assay in HEK293 cells expressing human ether-a-gogo related gene (HERG) K+ channels has classically been utilized to identify compounds that could possibly prolong QT (Bowlby et al., 2008). Recently, with the advent of stem cell-derived cardiomyocytes (CMs), cardiac functional assessments, such as multi-electrode arrays (MEA) (Ando et al., 2017; Yamazaki et al., 2018), patch clamps (Scheel et al., 2014), and calcium transient assays (Lu et al., 2015a; Pfeiffer et al., 2016), using human PSC-derived CMs have been utilized to predict drug-induced QT prolongation and fatal arrhythmia. Cardiac contractile function has also been assessed using non-invasive motion vector analysis or impedance-based measurements to predict drug-induced alterations in contractility (Hayakawa et al., 2012; Lamore et al., 2013; Mills et al., 2017; Poulton, 2017; Okai et al., 2020). Furthermore, HCA-based structural cardiotoxicity assays (Grimm et al., 2015; Sharma et al., 2017; Matsui et al., 2019), using cytotoxicity, sarcomere morphology, mitochondrial integrity, or reactive oxygen species (ROS) formation as readouts, have also been developed. These functional and structural assays have been exclusively developed using 2D monocultured stem cell-derived CMs. However, CMs occupy less than 50% of the whole heart (Bergmann et al., 2015) and, thus, their role cannot be neglected; additionally, the effects of drugs on non-cardiomyocytes and the subsequent indirect effects of non-cardiomyocytes on cardiomyocytes *via* cell-cell interactions cannot be assessed using the methods described.

Therefore, to further recapitulate cardiac physiological characteristics *in vitro*, various studies have been conducted to create 3D human cardiac organoids/microtissues using a cell suspension composed of stem cell-derived CMs) and various non-CMs [such as primary human cardiac fibroblasts (hCFs), human umbilical vein endothelial cells (HUVECs), primary human cardiac microvascular endothelial cells (hCMECs), and human adipose-derived stem cells (hADSCs)] that are seeded in hydrogel molds with ECM to assemble cardiac organoids (Patel et al., 2017; Richards et al., 2017; Patel and Birla, 2018; Mills et al., 2019; Schulze et al., 2019; Richards et al., 2020). Cardiac organoids/microtissues fabricated using these methods are expected to better reflect human heart physiology and be useful in modeling human cardiac disease, drug screening, and the development of regenerative medicines [44]. Unique 3D engineered cardiac tissues (ECTs) were developed using human hiPSC-CMs and fibroblasts in the scalable tissue-cultivation platform “BiowireTM II” that allows noninvasive recording of cardiac contractions, action potentials, and conduction velocities (Zhao et al., 2019). The research group embedded suspensions of both iPSC-CMs and cardiac fibroblasts in a hydrogel, then seeded them in a BiowireTM II platform microwell and conducted 10-weeks electrical field stimulations to promote maturation. Using the fabricated ECTs, they assessed the effects of several different positive and negative inotropes on cardiac contractility and obtained good concordance, suggesting that such assay platforms could be utilized early in the drug discovery process (Feric et al., 2019; Qu et al., 2020). A similar approach has also been developed by another group (Tiburcy et al., 2017; Cyganek et al., 2018). Interestingly, Li et al., for the first time, developed a novel *in vitro* system that allows for the direct measurement of cardiac performance as a pump by fabricating a 3D electromechanically coupled human ventricle-like cardiac organoid chamber (hvCOC) (Li et al., 2018; Keung et al., 2019). Using the hvCOC, they were able to measure detailed parameters related to cardiac contractility, such as stroke volume, EF, cardiac output, and pressure volume loop. Using the fabricated hvCOC and 25 cardioactive compounds covering various drug classes, they assessed the predictivity of the assay and achieved a high prediction accuracy of more than 80–100%. Furthermore, Archer et al. created 3D cardiac microtissues using cell suspensions composed of human iPSC-CMs, hCMECs, and hCFs, and seeded them onto 384-well ultra-low attachment spheroid microplates (Archer et al., 2018). Using the fabricated cardiac microtissues and HCA-based simultaneous detection of multiple parameters related to cellular damage (ATP depletion, endoplasmic reticulum (ER) integrity, and mitochondrial membrane potential), they assessed 15 FDA-approved structural cardiotoxins and 14 FDA-approved nonstructural cardiotoxins. The results showed that structural cardiotoxicity could be detected at therapeutically relevant concentrations and conferred high prediction accuracy when compared to 2D-cultured human iPSCs. Notably, *in vitro* modeling of post-myocardial infarction (MI) was performed by Richards et al. using cardiac organoids (Richards et al., 2020). They prepared cell suspensions containing 50% human iPSC-CMs and 50% non-myocytes, including hCFs, HUVECs, and hADSCs, seeded them into agarose hydrogel molds, and submerged them with culture medium for 4 days to create self-assembled organoids. Then, the organoids were incubated under hypoxic (10% O_2_) conditions and noradrenaline was added for 10 days to mimic the *in vivo* MI conditions in which there is compensatory activation of the sympathetic nerves to restore decreased cardiac output. Both functional and structural assessments as well as global gene expression analyses have demonstrated that the fabricated organoids can mimic many of the pathological hallmarks of MI (pathological metabolic shifts, increased fibrosis, and abnormal calcium handling). Enhanced doxorubicin-cardiotoxicity in the MI organoid model has also been reported. These findings suggest that human organoids modeling diseases with non-genetic pathologic factors are useful for drug screening and increase the possibility of personalized models for safety assessments.

### Limitations and Future Perspectives

3D cardiac organoids/microtissues generated from human PSCs do not yet contain all the cells present in the adult heart, such as immune cells, which play an important role in providing the protective inflammatory responses required for host defenses against infection (Lafuse et al., 2020); additionally, they are also reported to contribute to the pathogenesis of HF (Carrillo-Salinas et al., 2019). Thus, a novel assay system, such as the co-culture of PSC-CMs and immune cells, may help us to gain a deeper understanding of the crosstalk between CMs and immune cells. Furthermore, efforts to develop such assay systems may help reveal the mechanisms underlying immune checkpoint inhibitor-related cardiotoxicity (Michel et al., 2019; Behravesh et al., 2020; Xu et al., 2021) and to mimic such adverse effects *in vitro*. Another limitation of the 3D cardiac organoids/microtissues is that the degree of maturation for the fabricated organoids is not yet sufficient, when compared to adult cardiac tissues, in terms of morphology, function, and gene expression, and this prevents accurate predictions of drug-induced cardiotoxicity. To overcome this limitation, various strategies have been developed to enhance the maturation of PSC-derived CMs, such as the addition of hormones, changing cellular energy sources or substrate stiffness, conducting continuous electrical stimulations, and utilizing 3D culture systems (Ahmed et al., 2020). Such efforts toward maturation will contribute to a higher accuracy in predicting drug-induced cardiotoxicity in humans. Furthermore, from a practical perspective, each *in vitro* cardiotoxic assay should be used at different stages depending on the purpose during development (for early-stage screening or mechanistic toxicity study). A robust and standardized assay protocol is also required to minimize interfacility differences, especially when sophisticated *in vitro* assays are used for the mechanistic understanding of drug-induced cardiotoxicity.

## Kidney Organoids and the Prediction of Kidney Toxicity

### Overview

Drug-induced kidney injury (DIKI) is a serious problem worldwide (Pavkovic and Vaidya, 2016) and is responsible for 19% of acute renal failure cases (Cook et al., 2014). DIKI is also one of the causes of drug attrition during drug development (Cook et al., 2014). A wide variety of drugs, including cisplatin, NSAIDs, antibiotics, and contrast agents, are known to cause injury in various regions of the kidney (Soo et al., 2018). Therefore, *in vitro* assays that can predict drug-induced kidney injury during the early stages of drug development are urgently required. An ideal renal cell culture model would be one in which cells display the expression of functional drug transporters, the maintenance of metabolic functions, and the expression of biomarkers observed in clinical settings (Zhang and Parikh, 2019). These factors are expected to increase the value of DIKI predictions. However, in the absence of such ideal and appropriate renal cells, no standard or established *in vitro* assays have yet been used to predict DIKI. To date, 2D cultured human renal proximal tubule epithelial cells (PTECs) have been commonly used for the prediction of DIKI (Adler et al., 2016). This is because the proximal tubules are the primary target for DIKI, as they are most frequently exposed to drugs and have high metabolic activities, and imbalances in uptake and efflux activities cause intracellular accumulation of drugs and subsequent toxic injury. Su et al. evaluated a reference set of 44 compounds, including nephrotoxicants and non-nephrotoxicants, using 2D-cultured PTECs and high-throughput imaging analysis (Adler et al., 2016). With the use of machine learning techniques, the assay exhibited good predictability with an accuracy of more than 80%. PTEC has limited proliferative capacity and inter-donor variability in toxic readouts; thus, immortalized proximal tubule epithelial cell lines are also used to overcome these limitations. Another group also reported a similar HCA-based approach using conditionally immortalized PT epithelial cells that were stably expressing OAT1 for a panel of 62 drugs and they showed a good prediction accuracy with 75% sensitivity and 100% specificity (Sjogren et al., 2018). However, these 2D monoculture assays using PTEC or immortalized cell lines suffer from dedifferentiation, decreased expression of transporters, and decreased metabolic function. In addition, the kidney is a complex, multicellular organ composed of over 20 different cell types, and the parts of the nephron (glomeruli, proximal tubule, distal tubule, and collecting duct) that are affected varies with different nephrotoxic compounds, and there is a marked diversity in the mechanisms by which compounds affect renal function (Soo et al., 2018).

To address this issue, the creation of kidney organoids or kidney tissue models has become a focus to help mimic kidney physiology. In 2013, Desrochers et al. first reported the potential utility of a 3D human renal tissue system created using the transwell-based 3D cultures of immortalized human renal cortical NKi-2 cells embedded in an ECM solution (DesRochers et al., 2013). They evaluated the acute and chronic effects of representative nephrotoxic drugs (cisplatin, gentamicin, and doxorubicin) using 2D monocultured NKi-2 and 3D kidney tissue models and showed that 3D tissues were more sensitive to drug-induced toxicity. In 2015, three different groups reported fabrication methods for 3D kidney organoids from human stem cells. Freedman et al. reported the fabrication of segmented, nephron-like kidney organoids containing proximal tubule, podocyte and endothelial cell populations by adding a GSK3b inhibitor to cavitated spheroids formed by 3D cultured human stem cells; additionally, they also examined the reactivity of cisplatin and gentamicin in tubular organoids and confirmed elevated levels of Kim-1 (Freedman et al., 2015). Furthermore, in a separate study, they also reported a fully automated and high-throughput screening (HTS)-enabled platform for the production of kidney organoids from human PSCs, their analysis for toxicity assessment and improvement of mechanistic understanding of polycystic kidney disease (Czerniecki et al., 2018). Morizane et al. reported an efficient chemically defined protocol to differentiate human stem cells into nephron progenitor cells (NPCs) that can self-organize into nephron-like structures. They showed elevated expression of the KIM1 protein in the fabricated organoids upon simulation with gentamicin and cisplatin (Morizane et al., 2015). Takasato et al. differentiated human iPSCs not to nephron progenitor cells but to the intermediate mesoderm so that kidney organoids were made from two types of kidney progenitor cells. As a result, the kidney organoids contained not only tubules and glomeruli but also all cells necessary for kidney formation, including collecting ducts, blood vessels, and renal interstitial cells. The fabricated organoids showed an improved response to cisplatin, a nephrotoxicant that induces apoptosis in proximal tubular cells, suggesting that functional maturation of the nephrons within the fabricated organoids was provided (Takasato et al., 2015). To enable more accurate predictions of DIKI, King et al. created an *in vitro* model of the proximal tubule interstitial interface comprising renal fibroblasts, endothelial cells, and human PTECs using their proprietary 3D bioprinting platform (King et al., 2017). The fabricated 3D proximal tubule tissues demonstrated tight junction formation and expression of renal uptake and efflux transporters. Functional assessments revealed dose-dependent cisplatin toxicity, and that the OCT-2 inhibitor cimetidine rescued the injury and fibrotic response to TGFb (King et al., 2017). These 3D kidney organoids provide valuable information for the prediction of drug-induced kidney toxicity in humans.

### Limitations and Future Perspectives

Although the number of kidney organoid studies has gradually increased in previous years, there have been no reports in which a large number of drugs were tested in 3D kidney organoids, as has been the case for 2D cultures (Adler et al., 2016; Sjogren et al., 2018). To incorporate a predictive toxicity assay using kidney organoids for early screening during development, further comparative studies regarding the prediction accuracy between conventional 2D cultures and 3D organoid systems are required. In addition, *in vitro* toxicity assays have been performed exclusively based on the endpoints related to cell death, but incorporating appropriate readouts related to potential clinical DIKI biomarkers (KIM1, NGAL, and IGFB3) (Zhang and Parikh, 2019) may improve the prediction accuracy of DIKI in the 3D organoids. One drawback of 3D kidney organoids is the lack of blood flow; as the kidney is an organ that carries a large amount of blood, understanding its vascular structure is essential to accurately recapitulate kidney function. To avoid this problem, the usefulness of the Kidney-on-a-chip has been proposed (Wilmer et al., 2016). The ability to add shear stress has been shown to increase transporter expression (Vormann et al., 2018; Vriend et al., 2018). However, because these complex assay systems have poor throughput, it is necessary to determine the stage during drug development during which they be best utilized. It is also important to consider that various drugs are known to cause kidney injury in different parts of the nephron and the drugs currently used primarily for toxicity assessment using kidney organoids are those known to cause tubular toxicity rather than glomerular toxicity, such as gentamicin and cisplatin (Soo et al., 2018). Therefore, it is also essential to investigate if currently reported kidney organoids can also predict glomerular toxicity caused by drugs such as mitomycin C and nonsteroidal anti-inflammatory drugs (Markowitz et al., 2015) to further our understanding of the role of kidney organoids in predicting DIKI. Moreover, as there are no standard *in vitro* assays for DIKI, it is important to select an appropriate *in vitro* assay (high-throughput 2D monoculture or sophisticated 3D organoid) depending on the stage of drug development. Further efforts are needed to develop novel *in vitro* assay systems that better reflect the physiological functions of the human kidney.

## Intestinal Organoids and the Prediction of Gastrointestinal Toxicity

### Overview

Drug-induced gastrointestinal toxicity (Pusztaszeri et al., 2007) is characterized by abdominal discomfort, abnormal peristalsis, anorexia, nausea, vomiting, abdominal pain, abnormal defecation (diarrhea/constipation), abnormal stools (mucous, tarry, or whitish), flatulence, hematemesis, and melena, and these symptoms are often present in combination. It is a common adverse event and a reason for discontinuation during drug development (Cook et al., 2014). A wide variety of drugs are known to affect different parts of the gastrointestinal (GI) tract (Pusztaszeri et al., 2007; Carr et al., 2017). To model the *in vitro* upper and lower gastrointestinal tracts, several human GI cells are available, including cancer cell lines, immortalized cells, and primary cells (Carr et al., 2017). However, primary cells cannot be cultured for a long period of time (Grossmann et al., 1998) and cancer cell lines and immortalized cells have low metabolic activity, lose physiological functions upon passage, different gene expression profiles when compared to those *in vivo*, and no original functional properties. As a result, *in vitro* drug-induced intestinal toxicity screening is rarely performed when compared to the organ toxicity described in the previous sections. As a cell-based assay, Caco-2 is regarded as a surrogate enterocyte and has been used primarily as the gold standard in a number of absorption, distribution, metabolism, and excretion (ADME) studies, including drug absorption and metabolism assessments (Wang et al., 2016); however, it has been used in relatively few studies for the prediction of clinically reported gastrointestinal toxicity. In conclusion, the prediction of gastrointestinal toxicity in humans is entirely dependent on animal studies. However, due to the differences in anatomy, biochemistry, and microorganisms in the gastrointestinal tissues of species, and to avoid the cost and efforts required for animal studies, a highly predictive *in vitro* assessment system is required.

Recently, fabrication methods for human intestinal organoids from intestinal epithelial stem cells (Sato et al., 2009) or PSCs (Spence et al., 2011; Takahashi et al., 2018) have been reported. In 2009, Sato et al. showed for the first time that intestinal epithelial tissue organoids could be constructed from Lgr5-positive stem cells isolated from crypt regions in the small intestine (Sato et al., 2009). They showed that crypt regions, including Lgr5-positive stem cells suspended in Matrigel, allowed for the *ex vivo* reconstruction of intestinal tissues that reflected intestinal epithelial cell proliferation and differentiation. They also demonstrated that the fabricated intestinal organoids could be cultured for more than 8 months. In 2011, Spence et al. differentiated human PSCs into endoderm with activin A and then differentiated them into CDX2-positive intestinal endoderm by adding FGF4 and Wnt3A, and transferred the floating spheroids formed into 3D cultures by embedding them in Matrigel to promote intestinal growth and differentiation (Spence et al., 2011). The fabricated 3D intestinal organoids were composed of a polarized columnar epithelium that was patterned into villus-like structures and crypt-like proliferative zones. Takahashi et al. established a sophisticated protocol to produce intestinal organoids from human intestinal crypts isolated from the healthy intestinal tissues of surgical specimens without the use of cytokines and Matrigel that are generally required for the culture of 3D organoids in order to provide a suitable method for high-throughput screening (Takahashi et al., 2018).

These intestinal organoids have attracted attention for a variety of applications, including disease modeling, intestinal-microbe interactions, pharmacology, and regenerative medicine, and they have shown promise for the prediction of toxicity (Bitar and Zakhem, 2016; Dedhia et al., 2016; Almeqdadi et al., 2019). From a toxicological point of view, Belair et al. generated 3D spherical ileal organoids from a Matrigel-embedded ileal crypt suspension extracted from commercially available human ileal tissues (Belair et al., 2020). Using ileal organoids, they evaluated 31 marketed drugs known to cause different degrees of clinical incidence of diarrhea with cell viability as a toxicity readout to predict drug-induced diarrhea; they obtained 90% prediction accuracy (Belair et al., 2020). Peters et al. developed a non-spherical 3D Epi-Intestinal tissue model by culturing human fibroblasts and primary human small intestinal epithelial cells in collagen conditions in a Transwell format under an air-liquid interface (ALI) (Peters et al., 2019). Instead of focusing on endpoints related to cytotoxicity, such as ATP and LDH, which are often used in toxicity studies, they focused on the barrier function of the 3D microtissue model, as it is one of the GI-specific functional readouts. They performed a gut barrier function assay (TEER measurements) following treatment with 31 drugs used clinically as a reference set. Results revealed that the 3D Epi-Intestinal tissue model showed higher predictability than the 2D-cultured Caco-2 cells (Peters et al., 2019). These intestinal organoids/models appear to be promising for the prediction of drug-induced intestinal toxicity in humans.

### Limitations and Future Perspectives

3D intestinal organoids have attracted much attention in previous years, as they are expected to better reflect biological functions than conventional 2D cultures. However, currently reported 3D intestinal organoids do not contain cells associated with the vasculature, immune system, or enteric nervous system. In addition to diarrhea, drug-induced intestinal toxicity has a broad range of symptoms, and there are no models yet to predict nausea, vomiting, and constipation. Unlike gastrointestinal epithelial cells, complex co-cultures (involvement of the innate immune system and nervous system) may be required to further mimic gastrointestinal physiology. In addition, these 3D organoids are cultured under static conditions and do not experience fluid flows and cyclic mechanical deformations that are similar to physiological intestinal peristaltic movement. Thus, incorporating microphysiological systems, including organ-on-a-chip technology, into 3D intestinal organoids is expected to provide further potential value to recapitulate living human intestine (Bein et al., 2018). Indeed, Kasendra et al. developed a primary human small intestine-on-a-chip using biopsy-derived organoids (Kasendra et al., 2018). Global gene expression analysis revealed that organoids cultured on the intestine chip more closely mimicked the living duodenum than static duodenal organoids. It should also be taken into account that, in contrast to other organ disorders, there are relatively few validated biomarkers available for gastrointestinal diseases. As mentioned in the previous sections, the identification and validation of novel biomarkers related to gastrointestinal diseases and incorporating them as toxicity readouts will increase the predictive accuracy of *in vitro* assays using intestinal organoids. Furthermore, the fabrication of physiologically relevant intestinal organoids and cocultures with microbes (Puschhof et al., 2021) will provide a better understanding of host-microorganism interactions as well as drug-gut microbiota interactions that may be associated with altered therapeutic and toxic effects of various drugs (Weersma et al., 2020).

## Brain Organoids and the Prediction of Neurotoxicity

### Overview

Drug-induced neurotoxicity includes both the structural neurotoxicity associated with tissue damage and functional neurotoxicity, which includes seizure/convulsions (Walker et al., 2018). These adverse effects are the major causes for discontinuation in candidate drug development. It was reported that the percentage of discontinued projects due to CNS adverse effects was 7% in the preclinical stage and 34% in the clinical trial stage (Cook et al., 2014). Therefore, as in the case of the liver and heart, there is a requirement for the early identification of drug candidates that have a risk of neurotoxicity. Since the discovery of human iPSCs and the development of methods for their differentiation into neuronal cells, *in vitro* predictive assays using iPSC-neurons have been conducted in previous years. Specifically, to predict drug-induced structural and functional toxicity in humans, various readouts such as neuronal cell death, neurite outgrowth, calcium oscillation, and extracellular field potentials were measured in 2D cultured iPSC-derived neurons (Wilson et al., 2014; Bitar and Zakhem, 2016; Dedhia et al., 2016; Takahashi et al., 2018; Almeqdadi et al., 2019; Belair et al., 2020; Shirakawa and Suzuki, 2020).

In 2013, Lancaster et al. first reported the creation of 3D self-organising cerebral organoids from human stem cells (Lancaster et al., 2013). They generated neuroectoderm from human stem cell-induced embryoid bodies, embedded them in Matrigel, and transferred them to a spinning bioreactor to enhance nutrient absorption. After 20–30 days, 3D self-organizing cerebral organoids that exhibited various cell lineages, including the forebrain, midbrain, hindbrain, retina, and choroid plexus, were obtained. In addition, by altering the concentrations and compositions of the morphogen (external patterning factors) during culture, the stem cells could differentiate into brain region-specific organoids, such as the cerebral cortex (Pasca et al., 2015), hippocampus (Sakaguchi et al., 2015), midbrain (Qian et al., 2016), and cerebellum (Muguruma et al., 2015). Among them, cerebral brain organoids have attracted particular attention. Human brain organoids are expected to provide valuable information to improve our understanding of the mechanisms of neurodevelopmental or neurodegenerative disorders and are useful tools for modeling and drug screening for such diseases (Chhibber et al., 2020). In addition, 3D brain organoids/models have been used in previous years to understand the effects of marketed drugs or environmental toxicants on human neuronal development. Focusing on the fact that human cerebral organoids from iPSCs are similar to fetal brains in terms of development and structure, Arzua et al. modeled alcohol-induced neurotoxicity using human 3D cerebral organoids to understand the mechanisms of fetal alcohol spectrum disorders (Arzua et al., 2020). They cultured human iPSCs in a chemically defined medium to aggregate them into embryoid bodies in ultra-low attachment plates and to initiate differentiation into neuroepithelial tissues, followed by the formation of cerebral organoids *via* Matrigel embedding. They, then, exposed the organoids to ethanol and found increased caspase three activity and functional (OCR reduction) and morphological alterations (less dense matrix and disrupted cristae) of the mitochondria in the 3D cerebral organoids. Specifically, neurons are more vulnerable to alcohol-induced apoptosis than astrocytes. Bu et al. also fabricated 3D cerebral organoids from hESCs. They added acrylamide (ACR), which is a common food contaminant that has been reported to have neurotoxic effects in preclinical studies, to organoids to understand the mechanisms of ACR-induced neurotoxicity in human brain development (Bu et al., 2020). The results revealed significantly increased nuclear factor erythroid 2-related factor 2 (NRF2)-mediated gene expression, induction of cell apoptosis, repression of neuronal differentiation, and promotion of tau hyperphosphorylation following ACR exposure to brain organoids. Liu et al. examined the utility of human cerebral organoids for toxicity assessment using vincristine (a microtubule-destabilizing drug for cancer treatment) to understand the mechanisms of neurotoxicity or developmental neurotoxicity (Liu et al., 2019). They created organoids from human iPSCs using an approach similar to that used by Lancaster et al. (2013). After 48 h of treatment with vincristine, dose-dependent neurotoxicity and the inhibition of fibronectin and tubulin development were observed in the cerebral organoid (Liu et al., 2019). Pamies et al. proposed the utility of a human iPSC-derived 3D brain model for understanding drug-induced neurotoxicity and developmental neurotoxicity. The fabricated 3D models comprised differentiated mature neurons and glial cells (astrocytes and oligodendrocytes) to recapitulate neuronal-glial interactions (Pamies et al., 2018). They examined the effect of acute (24 h or 48 h) exposure to rotenone (a natural pesticide and inhibitor of complex I) using a 3D brain model and found that the toxicity of rotenone varied depending on the differentiation status of the cells, showing higher reactive oxygen species (ROS) and higher mitochondrial dysfunction during the early (2 weeks) rather than the later differentiation stages (4 or 8 weeks). Interestingly, dopaminergic-neuron selective toxicity was observed at low (non-cytotoxic) concentrations, while astrocytes and other neuronal cell types were affected at high (cytotoxic) concentrations. These reports suggest that 3D brain organids/models can be used to assess the toxicity of drugs and help us to gain a better understanding of the mechanisms responsible for toxicity.

### Limitations and Future Perspectives

There are some limitations inherent to 3D brain organoids, such as the absence of microglia and vasculature. To address some of these limitations, Cakir et al. designed hESCs to ectopically expressed human ETS mutant 2 (ETV2) and generated human cortical organoids (hCO) (Cakir et al., 2019). ETV2-expressing cells in hCO contributed to the formation of a complex vascular-like network in the hCO. Interestingly, the presence of vascular-like structures led to enhanced functional maturation of organoids. Several blood-brain barrier characteristics were also observed, including tight junctions, nutrient transporters, and increased expression of transendothelial electrical resistance. Such vascularized organoids will more faithfully recapitulate physiological functions and provide a valuable platform to better understand the mechanisms of brain development and disease, as well as to conduct drug screening *in vitro*. In addition, in the above-mentioned reports on human brain organoids, only a single drug or substance has been tested in a toxicity validation studies, and many drugs still need to be evaluated. To conduct safety screening in the early stages of drug discovery, whether the predictive accuracy of 3D organoid-based assays is superior to conventional 2D cultured assays should be urgently examined using a wide variety of currently marketed drugs. In addition, as described in previous sections, since the preparation of 3D brain organoids requires multiple steps and a significant investment of time, attention should be paid to the reproducibility of results among studies based on organoids, as well as to the assay throughput.

## Concluding Remarks

In this review, we summarized the latest information on human organoids from a toxicological perspective and discussed their usefulness, limitations, and prospects, focusing on possible *in vitro* predictive assays for major organ toxicities (liver, heart, kidney, gut, and brain) that are caused by marketed drugs. There is currently no standardized method for predicting drug-induced organ toxicities using human organoids, and the number of reports describing toxicity studies using human organoids is still very limited. However, as organoid research has shown remarkable progress in the past 10 years and the number of studies on human organoids has increased, more studies are expected in the future to show the potential usefulness of organoids for toxicity evaluations. Furthermore, the combination of 3D organoid cultures and genome editing (Clevers, 2016), organs-on-a-chip technique (Yu et al., 2019) and further attempts to promote the maturation of organoids will help more accurately model human genetic diseases and create more physiologically relevant *in vitro* models of human organs. These efforts will allow for a better understanding of disease mechanisms, to perform highly predictive assays for drug-induced toxicity, and select better drug candidates during development.

## Abbreviations

ASCs, adult tissue-resident stem cells; ADME, absorption, distribution, metabolism, and excretion; CMs, cardiomyocytes; DIKI, drug-induced kidney injury; ECTs, engineered cardiac tissues; ETV2, ETS mutant 2; hADSCs, human adipose-derived stem cells; hCFs, human cardiac fibroblasts; hCMECs, human cardiac microvascular endothelial cells; hCO, human cortical organoids; HUVECs, human umbilical vein endothelial cells; hvCOC, human ventricle-like cardiac organoid chamber; iPSCs, induced pluripotent stem cells; MEA, multi-electrode arrays; PL, phospholipidosis; PSCs, pluripotent stem cells; PHH, primary human hepatocytes; PTECs, proximal tubule epithelial cells.
